# Vehicle-to-infrastructure IEEE 802.11ad Wi-Fi dataset

**DOI:** 10.1016/j.dib.2023.108964

**Published:** 2023-02-09

**Authors:** Mateus Mattos, António Rodrigues, Rui Meireles, Ana Aguiar

**Affiliations:** a Instituto de Telecomunicações, FEUP DEEC, University of Porto, Portugal; b Computer Science Department, Vassar College, USA

**Keywords:** VANET, V2I, mm-Wave, 802.11ad, Antenna sector selection, Directional communication

## Abstract

Despite not being designed for vehicular use, the high bandwidth offered by IEEE 802.11ad makes it an enticing proposition for opportunistic Vehicle-to-Infrastructure (V2I) communication.

Because it operates at a high frequency of 60 GHz, 802.11ad suffers from high attenuation. To combat this, it uses antenna directionality to improve communication range. Directionality is primarily achieved by selecting an antenna configuration, or sector, from a list of preconfigured ones.

Choosing a good antenna sector is difficult in V2I environments, as the fast mobility involved affects the alignment between communicating nodes.

This article describes a dataset that supports analysis of Commercial Off-The-Shelf (COTS) 802.11ad device behavior in an experimental V2I communication scenario. More specifically, the dataset summarizes the results from a set of experiments in which a mobile client drove around a stationary Access Point (AP) while downloading data from it. Information regarding the client's mobility, control frames exchanged, and achieved throughput was collected.

This dataset can support realistic analysis of 802.11ad COTS equipment behaviors, such as antenna selection and communication range, in a vehicular communication scenario.

Specifications table

**Table:** 

Subject	Computer Networks and Communications

Specific subject area	Vehicular wireless networks
Type of data	Table
How the data were acquired	Pseudorandom data was sent from a stationary Access Point (AP) to a mobile client over 802.11ad. Frames were collected with the *tcpdump* utility by a monitor device collocated with the mobile client. Hardware used: (i) Three TP-Link Talon AD7200 routers [2] (one used as stationary AP, another as mobile client, and a third as monitor), running the LEDE-AD7200 Operating System [3]; (ii) Trimble 6H Pro Series GPS receiver [4], installed on the mobile client.
Data format	Filtered
Description of data collection	The AP vehicle was parked at the corner of a residential intersection. It then started sending data to the client, while the latter drove a circuit around the intersection, traversing it in all possible (approach direction, departure direction) combinations. Mobile client speeds varied between 0 and 50 Km/h, or 14 m/s.
Data source location	· City/Town/Region: Vila Nova de Gaia · Country: Portugal · Latitude and longitude: 41.111929 N, 8.631083 W
Data accessibility	Dataset: • Repository name: Zenodo • DOI: 10.5281/zenodo.7026550 • Direct link to data: https://doi.org/10.5281/zenodo.7026550 Code used to collect dataset: • Repository name: Zenodo • DOI: 10.5281/zenodo.7259330 • Direct link to data: https://doi.org/10.5281/zenodo.7259330
Related research article	Mateus Mattos, António Rodrigues, Rui Meireles, Ana Aguiar, Geolocation-based Sector Selection for Vehicle-to-Infrastructure 802.11ad Communication, Elsevier J. of Computer Communications, 193 (2022), pp. 224-233. https://doi.org/10.1016/j.comcom.2022.07.005.

## Value of the data

• **Importance:** The provided experimental data provides insight into the behavior of COTS IEEE 802.11ad in the context of Vehicle-to-Infrastructure communication. There are two main reasons why this is important and useful. First, this is a novel use for IEEE 802.11ad, which was not designed with mobility in mind. Second, due to the logistics involved, the vehicular context is difficult to collect experimental data for.
• **Target audience:** The provided dataset provides utility to researchers in the vehicular wireless communications space, particularly those who lack the resources to conduct their own vehicular experiments, or those looking to compare results from their experiments against others performed in different environments.
• **Future use:** The data was collected with the intent to support the analysis of both off-the-shelf and custom 802.11ad antenna sector selection algorithms in vehicular contexts, as exemplified by Mattos *et al.* [1]. It can support further study in this area, as it contains very detailed data (e.g., raw control frames) that have not yet been fully explored. It can also be used to compare the behavior of the Talon AD7200 routers used with other COTS equipment used under similar scenarios. More generally, because the dataset features geographically indexed throughput information, it can also be used to study the feasibility of using IEEE 802.11ad for different vehicular communication applications, and the overall impact of mobility on IEEE 802.11ad communication.

## 1 Objective

The dataset was collected to support: i) the analysis of COTS IEEE 802.11ad device antenna sector selection behavior, and ii) the potential benefit of using geolocation as a heuristic for 802.11ad antenna sector selection. This work was published in the article “Geolocation-based Sector Selection for Vehicle-to-Infrastructure 802.11ad Communication” [1].

This data article contributes to the reproducibility of the related research paper – other researchers could redo the analysis, using the same exact data. Moreover, it allows researchers to expand upon the original analysis, as well as use it to study entirely different aspects of IEEE 802.11ad network behavior.

## 2 Data description

The dataset contains data from two sets of experiments, one performed in 2020, and another in 2021. The subfolders “2020” and “2021” contain the data from the experiments performed in the respective year. Each set of experiments is divided into traces. A trace represents an uninterrupted period of data collection where a particular mobility pattern was followed, and is identified by a unique integer number. The mobility pattern used for each trace is described in Zenodo [5].

Each of the experiment set subfolders contains the following files:

• “**gps.csv**”: client vehicle mobility data. It is a table where each row represents a NMEA (National Marine Electronics Association) sentence, containing PVT (Position-Velocity-Time) data obtained from the GPS receiver. Each NMEA sentence is annotated with its type (e.g., GPRMC), and the system's timestamp at the time of its reception. Each NMEA sentence contains a subset of time, location, altitude, speed, heading, and precision GPS information. Because each sentence contains only a subset of fields, merging of multiple sentences may be required to get a full view of the client's mobility. This merging can be performed using the GPS timestamp, which is present in all sentences.
• “**gps-merged.csv**”: summarized client vehicle mobility data, indexed by GPS timestamp. It is a summary table created from “gps.csv”, for ease of use. Each row contains location, altitude, speed, heading, precision GPS information, and system timestamp for a given GPS timestamp. The resolution is 1 Hz.
• “**thrghpt.csv**”: application throughput data. This is a table where each row represents the amount of data successfully transferred over a period of time (typically one second). Each row contains, among others, a system timestamp, the number of bytes received by the data consumer application between the previous entry's timestamp and this entry's timestamp, and the corresponding throughput, in Mbit/s.
• “**wifi.csv**”: a table that summarizes the 802.11ad control frames captured by the monitor router. It contains frames of types that were deemed of interest for studying antenna sector selection (the full list of frame types is listed in Zenodo [5]). Each row in this file is made up of a list of columns, each representing a field in the original frame. Columns include the system's timestamp upon capture, physical layer information such as the radio channel and data rate used, and Sector-Level-Sweep (SLS)-specific information such as the sweep's SNR (Signal-to-Noise Ratio) report.
• “pcap/*”: the raw “.pcap” files created by *tcpdump*, and used to generate “wifi.csv”, separated by trace.
• “**configs/tshark.json**”: *tshark* configuration file used to create “wifi.csv” from the raw “.pcap” files, in JSON (JavaScript Object Notation) format.

Zenodo [5] includes additional information concerning the tabular “.csv” files above, namely a description of each individual column.

## 3 Experimental design, materials and methods

The goal was to collect a dataset of 802.11ad sector selection and performance data in a realistic V2I communication scenario. For this purpose, an AP was deployed at the corner of an intersection and a client vehicle was driven around it, while downloading data from the AP.

To not disturb traffic, the experiments were performed in a quiet residential area in Vila Nova de Gaia, Portugal (GPS coordinates 41.111929 N, 8.631083 W). To assess the repeatability of the results, the experiments were performed twice. First in July 2020, and then again in October 2021. The weather was dry and clear on both days, and no major construction work was carried out in the area in the 15 months between the two campaigns.

**Fig. 1:**
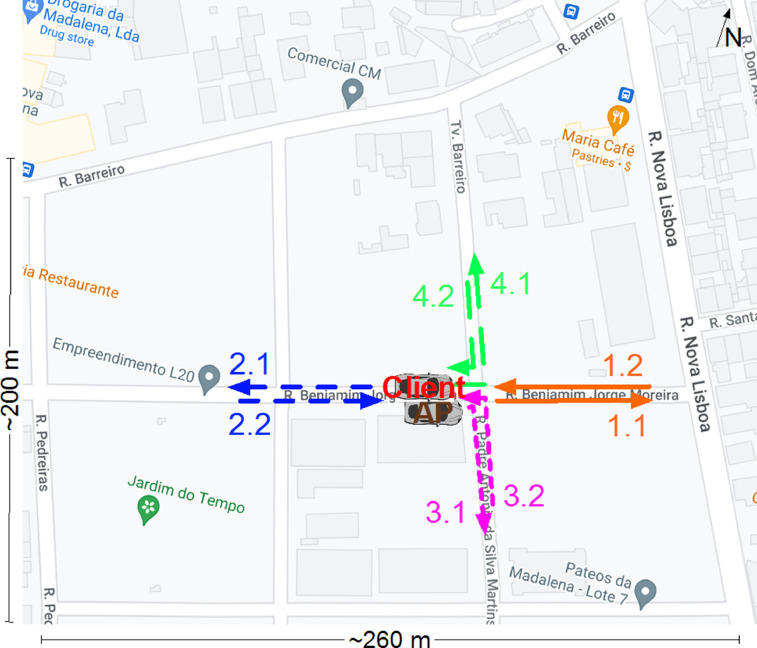
Slow speed client mobility pattern. Each experiment is numbered, and divided into two halves. For example, the first experiment consisted of moving eastwards away from the AP (1.1), and then back towards it (1.2). Map data ©2022 Google.

Two different types of mobility pattern were tested. In the first pattern the mobile client started next to the AP, slowly (i.e., at around 5 Km/h) moved away from it, in a straight line, until connectivity was lost, and then turned around and came back. The exercise was repeated for all 4 cardinal directions, as shown in Fig. 1.

This slow mobility pattern enabled the collection of spatially-dense performance measurements, i.e., a large number of samples per unit of spatial area in a short amount of time.

**Fig. 2:**
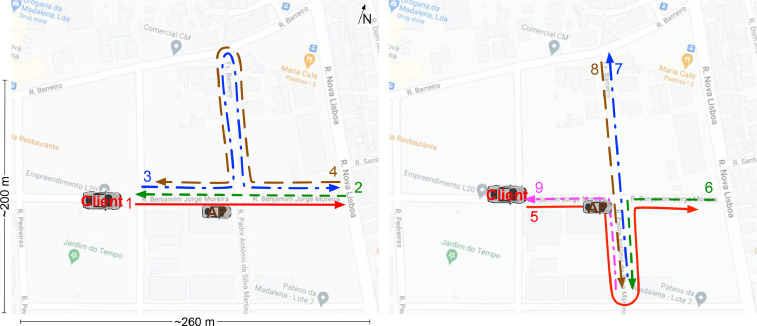
High speed client mobility pattern. The vehicle drove in a circuit. Each lap consisted of approaching the intersection from all possible directions, in the order listed. Map data ©2022 Google.

In the second pattern, the client vehicle drove a circuit around the intersection, such that it approached and departed from it in all possible direction combinations. Fig. 2 enumerates all the movements made by the vehicle in a single lap of the circuit.

Normal road speeds were used. The vehicle slowed down as it approached the intersection, to look for oncoming traffic, but then sped up to near the 50 Km/h legal limit as it drove away. This enabled the collection of data in a realistic setting for urban vehicular environments. This vehicle drove around this circuit for around 45 minutes in 2020, and another 45 minutes in 2021.

**Fig. 3:**
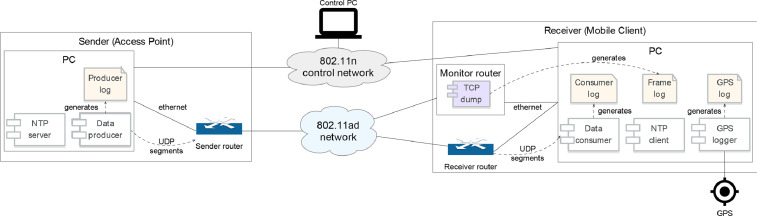
Data collection framework architecture. Modified from Mattos *et al*[1].

The software and hardware framework used in the experiments is now described. Its architecture is depicted in Fig. 3.

The system was comprised of two main nodes, a sender and a receiver. The sender node was made up of two devices: a TP-Link Talon AD7200 802.11ad router [2], and a laptop Personal Computer (PC), interconnected through Gigabit Ethernet.

The laptop ran a custom data producing application that generated pseudorandom data and sent it to the receiver's IP address, at the fastest possible rate, which in practice was around 420 Mbps. The amount of data generated was logged to a timestamped, 1 Hz-resolution, file. The data frames generated by the laptop were forwarded over Ethernet to the router, which then forwarded them over its 802.11ad wireless network. The router was set to Access Point mode, and used 802.11ad's channel #1 (60.48 GHz center frequency, 2160 MHz bandwidth). Since 802.11ad is theoretically capable of data rates of up to 8 Gbps, the Gigabit connection between laptop and router introduced a potential bottleneck. This was necessary because we were not able to run the data-producing application on the router itself. The reason is that the factory firmware does not support custom-code execution, and the open source alternative [3] often crashed when running in Access Point mode.

The mobility patterns we used had the client frequently going out of the AP's radio range, i.e., connectivity was intermittent. For this reason, Transmission Control Protocol's (TCP's) traditional flow and congestion controls would be troublesome. They equate losses with congestion, backing off the sender to alleviate it. When losses are caused by disconnection, upon reconnection the sender should resume sending at the same rate it was previously using. To circumvent this issue, we chose User Datagram Protocol (UDP) transport for the data transmitted between sender and receiver.

The composition of the receiver node mostly mirrored that of the sender node. There was a laptop, and a router used for 802.11ad communication. The laptop ran a data consuming application that worked as a sink for the data coming in from the sender node. It created a timestamped log recording how much data was received during each second. Additionally, since the receiver is also the mobile client, it was equipped with a high precision Trimble Pro Series 6H GPS receiver [4], to keep track of its movement. A 1 Hz-resolution log with all of the GPS data was created by a logger application.

Given the experiment's goals, capturing the Wi-Fi control frames pertaining to antenna sector selection was essential. These frames are ordinarily handled by the network interface and hence invisible to user-level processes running on the router. In order to make these frames visible, the network interface must be set to promiscuous mode, which lets all traffic through. However, a network interface so conFig.d cannot be used for regular communication. For this reason, a second Talon AD7200 802.11ad router was added to the receiver node, to act as a monitor.

The monitor router captured 802.11ad frames through the *tcpdump* utility. In order to be able to run *tcpdump*, the factory operating system was replaced with a version of LEDE with added 802.11ad support, created by the Talon Tools project [3]. Due to the router's storage limitations, the capture logs were stored on the laptop's drive, which was remote mounted using the Network File System (NFS) protocol. Ideally, there would be a monitor router on the sender side as well, but unfortunately no additional 802.11ad-capable routers were available. After collection, *tshark* was used to filter the frames and fields of interest. The frame types that were kept are specified in Zenodo [5].

To be able to match the producer log created by the sender with the logs created by the receiver, the system clocks on both laptops were synchronized through the Network Time Protocol (NTP). The server ran on the sender side, with the receiver being a client. NTP messages were exchanged using a separate 802.11n wireless control network. 802.11n provides a large communication range, important for uninterrupted connectivity. This control network was also used to orchestrate (i.e., start, stop, and monitor) the experiments from a separate control PC.

The code used in the experiments, along with usage instructions, is available to download from Zenodo [6].

**Fig. 4:**
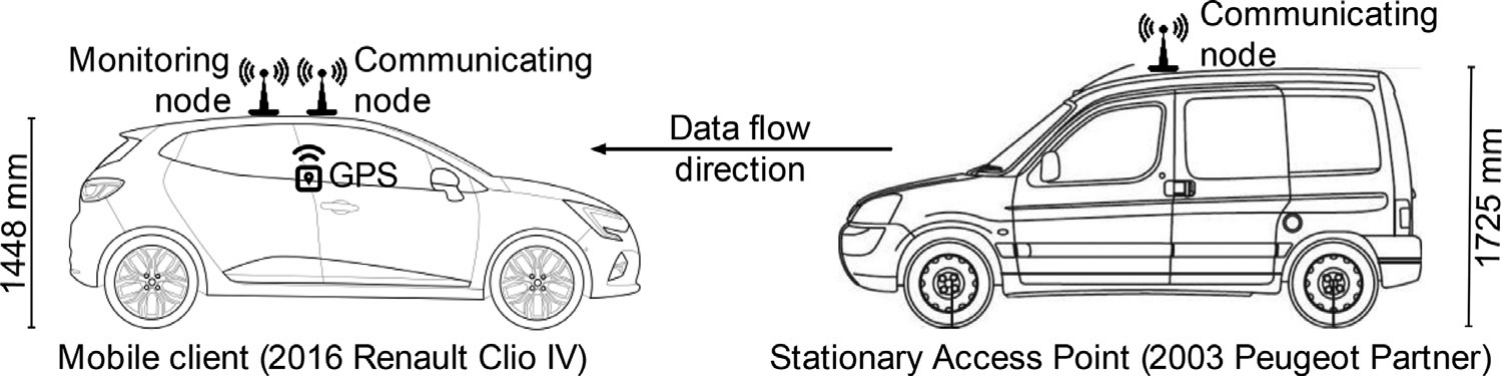
Data collection setup. Modified from Mattos *et al*[1].

Fig. 4 depicts the system's physical setup. Vehicles were used for both the mobile client and the AP. Even though the latter was stationary, the vehicle provided a convenient power source and mounting location. The communication devices were installed on top of the vehicles, to reduce the probability of line-of-sight obstructions as much as possible. Additionally, a small commercial van was used for the AP, in order to gain some additional height. The monitor router was placed right beside the communicating router, to minimize the difference between the channel conditions they experienced.

## Ethics statements

The work here presented did not involve human subjects, animal experiments, or any data collected from social media platforms.

## CRediT authorship contribution statement

**Mateus Mattos:** Conceptualization, Methodology, Software, Formal analysis. **António Rodrigues:** Conceptualization, Methodology, Validation, Data curation, Software, Formal analysis, Visualization, Writing – original draft. **Rui Meireles:** Conceptualization, Methodology, Validation, Software, Formal analysis, Visualization, Writing – original draft. **Ana Aguiar:** Conceptualization, Methodology, Supervision, Project administration, Funding acquisition, Resources, Writing – review & editing.

## Declaration of Competing Interest

The authors declare that they have no known competing financial interests or personal relationships that could have appeared to influence the work reported in this paper.

## Data Availability

Vehicle-to-Infrastructure IEEE 802.11ad Wi-Fi dataset (Original data) (Zenodo). Vehicular Wi-Fi Data collection framework source code (Original data) (Zenodo).
